# A Novel CD105 Determination System Based on an Ultrasensitive Bioelectrochemical Strategy with Pt Nanoparticles

**DOI:** 10.3390/s121013471

**Published:** 2012-10-08

**Authors:** Suyun Zeng, Sujun Wang, Li Wang, Lihua Yang, Zhenbo Chen, Zhiqing Liang

**Affiliations:** 1 Department of Obstetrics & Gynecology, Southwest Hospital, Third Military Medical University, 30 Gaotanyan Street, Shapingba District, Chongqing 400038, China; E-Mails: zengsuyun2012@126.com (S.Z.); baihe199166@qq.com (L.W.); ylh_960228@yeah.net (L.Y.); kennyawp@163.com (Z.C.); 2 Department of Biomedical engineering, Chongqing University, 174 Shazhengjie, Shapingba District, Chongqing 400044, China; E-Mail: bihaiwsj@126.com

**Keywords:** CD105, immunosensor, nanomaterial

## Abstract

CD105 is a well-known tumor metastasis marker and useful for early monitoring of metastasis and cancer relapse. It is important to generate rapid, reliable and precise analytical information regarding CD105 levels. To establish a simple, selective and sensitive detection method, we prepared an immunosensor with novel bioconjugates based on Pt nanoparticles, thionin acetate and antibodies. The proposed immunosensor displayed a broader linear response to CD105, with a working range of 1.3 to 200.0 ng/mL and a detection limit of 0.9 ng/mL under optimal conditions. Moreover, the studied immunosensor exhibited high sensitivity, fast analysis and adequate stability. The proposed methodology could readily be extended to other clinical- or environment-related biospecies.

## 1. Introduction

Metastases (MTS) resistant to conventional therapies are the major cause of death from cancer [1–3]. Early detection of tumor MTS is extremely important and has attracted considerable research interest [4]. Recently, several reports have shown that CD105 (EDG, endoglin), as a proliferation-associated and hypoxia-inducible protein, may be a good tumor MTS marker [5–11]. Some immunohistochemistry studies have revealed that CD105 is abundantly expressed in angiogenic endothelial cells of tumor tissues and shed into the circulation with elevated levels detectable in patients with various types of cancer and positively correlated with tumor metastasis. The potential usefulness of CD105 as a tumor MTS marker has been evaluated by Norihiko *et al.* [12]. These results suggested that serum CD105 may be a useful marker for monitoring early signs of MTS and cancer relapse in a long-term follow-up study of patients with solid tumors and other angiogenic diseases [13].

Conventional immunoassay methods for the detection of CD105 include radioimmunoassays (RIA) and enzyme-linked immunosorbant assays (ELISA). Electrochemical immunosensors have attracted great interest due to their potential utility as specific, simple, label-free and direct detection techniques with advantages that include reductions in size, cost and time of analysis [14]. Compared with conventional immunoassay techniques, electrochemical immunosensors exploit the coupling of highly specific recognition events between antibodies and antigens to appropriate transducers. Therefore, many kinds of electrochemical immunosensors have been developed. In particular, the advanced materials based on nanoparticles are currently one of the key research fields since they provide a larger surface area, good biocompartibility and stability on the electrode surface [15–17]. Recently, some groups have reported immunosensors based on gold nanoparticle (AuNP)-modified electrodes, which have good accuracy and long-term stability [18–20]. However, the selectivity of the resulting immunosensors was limited, as only one source of antibody to CD105 is currently available. It is probable that a sandwiched immunosensor with a second antibody would increase the selectivity of the immunosensor.

In this work, a detection immunosensor with capture antibodies (Ab1) to CD105 adsorbed on AuNP was obtained first. In order to increase the sensitivity and selectivity of the immunosensor, we prepared a second antibody (Ab2) that was chemically linked to the electron mediator, thionin acetate (THI), which was then adsorbed onto platinum nanoparticles (PtNP). The determination system was obtained via the Ab1 modified immunosensor and the PtNP-THI-Ab2.

## 2. Materials and Methods

### 2.1. Materials

Chloroauric acid, (hydro)chloroplatinic acid, ascorbic acid and bovine serum albumin (BSA) were purchased from Sigma Chemical (St. Louis, MO, USA). Sodium citrate was bought from Alfa Chemical (Beijing, China). All other reagents were analytical grade. All aqueous solutions were prepared with double-distilled water.

The AuNP was prepared by adding 2 mL of 1% (w/w) sodium citrate solution to 50 mL of 0.01% (w/w) HAuCl kept at 100 °C as described previously [18–20]. The PtNP was obtained by a similar method with a minor modification. The particle sizes were confirmed by scanning electron microscope (SEM).

CD105 is one kind of recombinant protein purified from prokaryotic cells, which have constructed a CD105 expression vector PET32a-CD105 in it. The detection pair of antibodies with first antibody (Ab) and Ab was obtained from mice using the purified CD105 proteins as immunization.

The PtNP, THI and Ab bioconjugates were prepared as follows. Firstly, the Ab was conjugated with THI by the reaction between –NH of THI and –CHO was oxidized from the –OH of Ab by potassium permanganate. Subsequently, 100 μL of PtNP solution was added in the mixture and incubated at 4 °C for 12 h, followed by centrifugation at 3,000 rpm at 4 °C for 20 min to remove non-activated PtNP and 12,000 rpm at 4 °C for 10 min to remove the PtNP-THI-Ab2 from excess reagents. Finally, 100 μL BSA was added to the complexes formed to block the unmodified portion on the PtNP. The obtained PtNP-THI-Ab2 bioconjugates was redispersed in 1 mL of PBS and stored at 4 °C when not in use.

### 2.2. Apparatus

Cyclic voltammetry (CV) measurements were performed with a CHI660d electrochemical workstation (Shanghai CH Instrusments, Shanghai, China). Bare or modified gold electrodes (4 mm in diameter) were used as the working electrode, a saturated calomel electrode (SCE) was used as the reference electrode and a platinum wire was used as the counter electrode. The working, reference and counter electrodes were used to form an electrochemical cell as the immunoassay system. All potentials are reported relative to the SCE reference electrode. SEM (Hitachi Co., Tokyo, Japan) was used to characterise the sizes and structures of AuNP and PtNP.

### 2.3. Preparation of the Immunosensor

**Figure 1.**
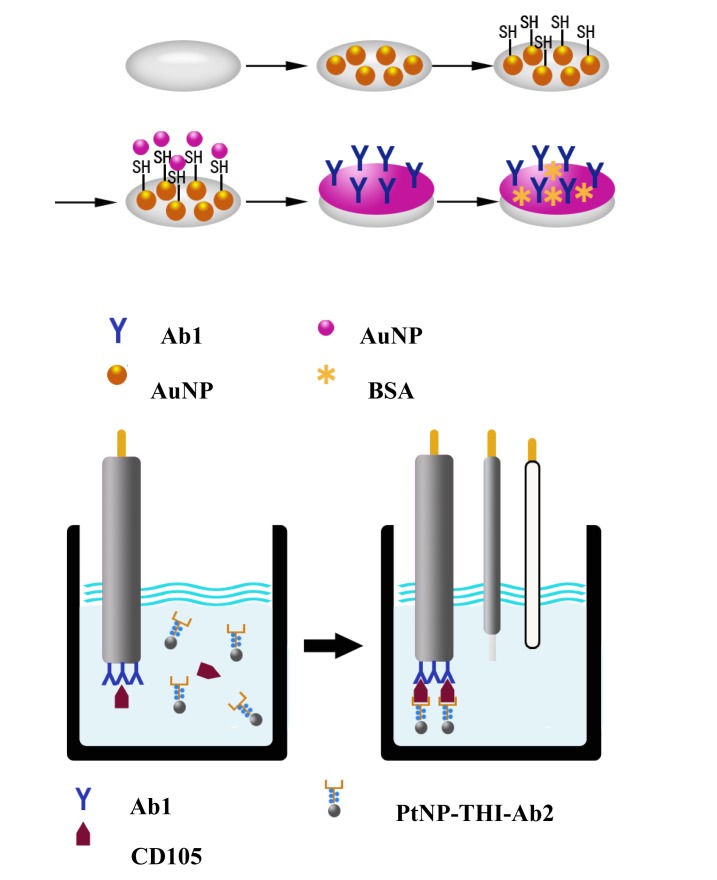
Diagram of the measurement and regeneration procedures of the proposed immunosensor.

The immunosensors were prepared as shown in the protocol schematic in Figure 1. Before the modification, the gold electrodes (GE) were polished carefully with alumina slurries (0.3, 0.05 μm). After the cleaning, the gold electrodes were ultrasonicated in acetone, water and ethanol, respectively. Then, the polished gold electrodes were dipped in a mixture of 1:1 HCl:H_2_O_2_ (v/v) for 10–15 s, rinsed with copious amounts of water and air dried to remove the remaining chemicals on the electrode surface. After cleaning the bare gold electrode, the AuNP was layed onto the electrode by applying a constant potential of −0.2 V for 60 s in HAuCl solution (0.01%, m/v) (AuNP/GE). After cleaning with water, the AuNP modified electrode was immersed in mercaptoethylamine solution for about 12 h to form a monolayer with –SH or –SS–(SN/AuNP/GE). Following that, the SN/AuNP/GE was dipped in the AuNP solution for 12 h to absorb the AuNP onto the surface of the electrode. Subsequently, the prepared electrodes were dipped in a solution containing the CD105 antibodies for 4 h and blocked by dipping in a BSA solution for 2 h. The finished electrodes were stored at 4 °C when not in use.

### 2.4. Experimental Measurements

All amperometric responses were obtained from CV detections in PBS solutions with 1.0 mM H_2_O_2_. In our experiments, detections were based on the changes in amperometric response with or without an antigen–antibody reaction. The steady-state amperometric responses were obtained without any immunochemical incubation in the black solution (*i*) and with immunochemical incubation in a standard CD105 solution (*i*). The amperometric response of the immunosensor to CD105 antigen was evaluated with the equation: Δ*i* = *i* − *i*.

## 3. Results and Discussion

### 3.1. Characterisation of the Nanomaterials by SEM and TEM

SEM and TEM are important tools to reveal the actual particle shapes of the modified electrode surface and hence are able to provide very useful data regarding the surface modification that occurs during the procedures described above.

**Figure 2.**
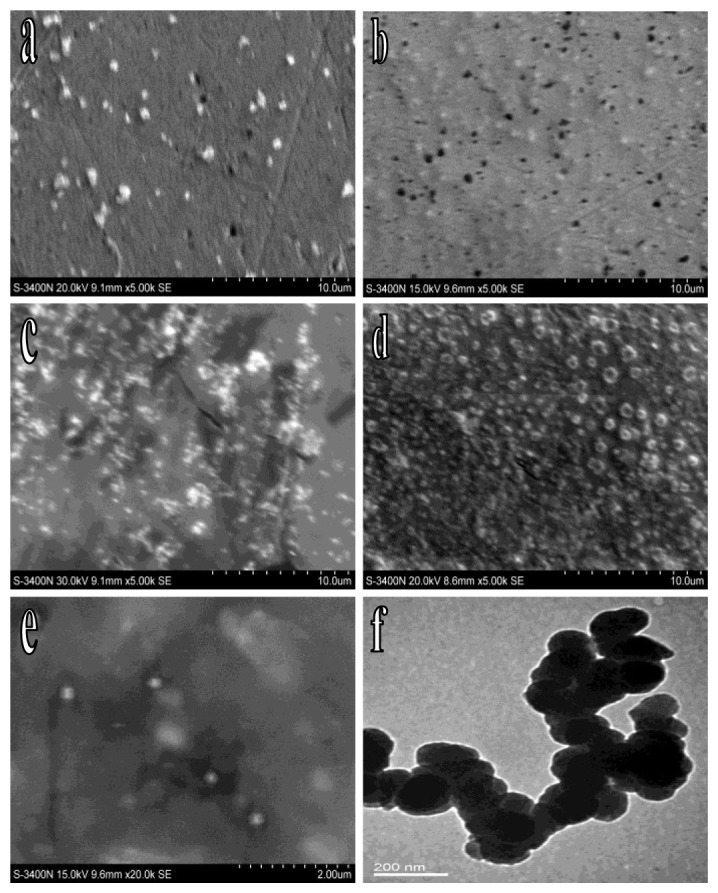
Characterization of the nanomaterials by SEM and TEM. SEM of AuNP (**a**); AuNP/SN (**b**); AuNP/SN/AuNP (**c**); AuNP/SN/AuNP/Ab @BSA (**d**); and PtNP (**e**); TEM of PtNP (**f**).

To study the modification of the immunosensor, SEM was used. As shown in Figure 2(a), the AuNP was uniformly dispersed on the surface of the electrode. After the modification of SN, a film could be seen (Figure 2(b)). The AuNP with smaller volumes and a relatively larger specific surface area could adsorb uniformly onto the electrode surface at a higher concentration (Figure 2(c)). When the antibodies adsorbed onto the AuNP surface to form a biological activity film the integration of the CD105 was more sensitive. The active sites remaining on the AuNP on the surface of the electrode was blocked with BSA to avoid any non-specific adsorption and a visible protein film could be seen on the electrode surface when examined by SEM (Figure 2(d)). To study the actual particle shapes of PtNP, the SEM and TEM were used simultaneously. As shown in the SEM image (Figure 2(e)), the PtNP was distributed evenly with a mean size of 200 nm. At the same time, the PtNP appeared to be with similar size and shape in the TEM image (Figure 2(f)).

### 3.2. Electrochemical Characterization of the Immunosensor

**Figure 3.**
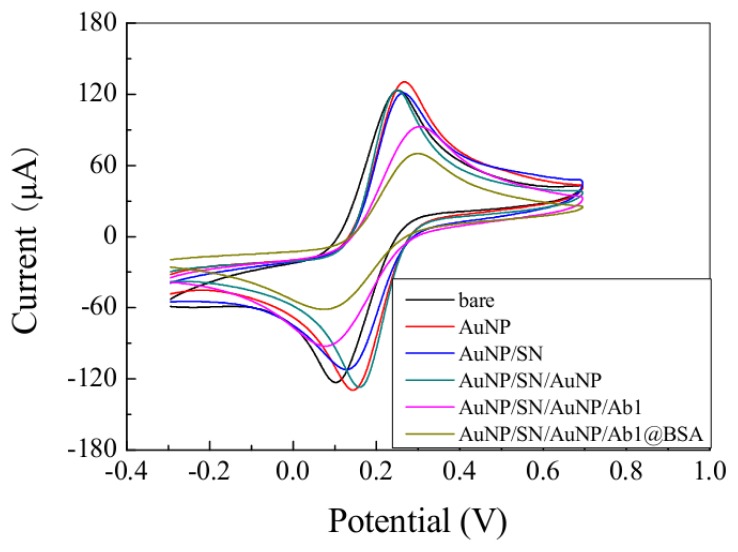
CVs of bare electrode (black curve) and electrodes modified with different surface: AuNP (red curve), AuNP/SN (blue curve), AuNP/SN/AuNP (green curve), AuNP/SN/AuNP/Ab1 (magenta curve), AuNP/SN/AuNP/Ab1@BSA (black yellow curve).

The electroactivities of the immunosensors based on CV were studied in a solution of 2 mmol/L [Fe(CN)_6_]^4−^/[Fe(CN)_6_]^3−^ (1:1; v/v) with 0.1 mol/L KCl, and the results are shown in Figure 3. The black curve shows the CV of the bare electrode. Firstly, an increase of current response could be seen after the electrode was modified with AuNP (red curve). As shown by the blue curve, the current response was decreased on the SN/AuNP modified electrode when compared with the AuNP modified electrode. After AuNP was adsorbed onto the SN/AuNP modified electrode (green curve), a predicted current increase could be seen which indicates that the NGP was immobilized on the electrode surface. When the electrodes were immersed with anti-CD105 (magenta curve) and blocked with BSA (dark yellow curve), the current clearly decreased. This is predictable because the antibodies and BSA should block the electron transfer. All of the results indicate that the modification processes were successful, and the proposed immunosensor could be used in the further detection of CD105.

### 3.3. Optimization of Assay Conditions

The influence of temperature on the current response was investigated at different temperatures ranging from 15 to 45 °C, under the same experimental conditions with 40 ng/mL of CD105. It was found that the current response increased with increasing temperature until 37 °C, and this leveled off above 37 °C, indicating the immunocomplexes may be destroyed over 37 °C. However, considering the activity of biomolecules and the life-time of biosensors, we opted for 25 ± 0.5 °C as the incubation temperature.

The effect of incubation pH on amperometric response was also investigated with pH values of 5.0 to 8.5 using 40 ng/mL of CD105 at 25 °C. The current response increased with increasing pH value from 5.0 to 6.5, then the current response decreased as pH value increased further and the maximum response was obtained at pH 6.5. It is well known that the activity of immunoproteins is inhibited at relatively low pH, so pH 6.5 was selected as the incubation pH for further studies.

The concentrations of H_2_O_2_ in the detection buffer were also evaluated by the method of using immunosensors, which has been incubated with 40 ng/mL of CD105, at pH 6.5 in PBS containing different concentrations of H_2_O_2_. When the H_2_O_2_ was 1.0 mM, the peak current response was achieved, therefore, 1.0 mM H_2_O_2_ was subsequently employed as the optimum concentration.

### 3.4. Calibration Plot for Determination of CD105

**Figure 4.**
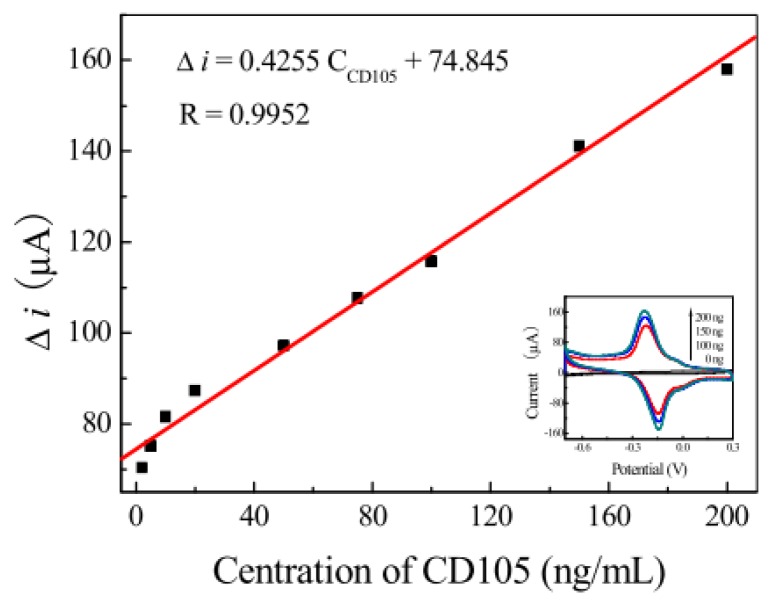
The current responses of the proposed immunosensor to CD105 with different concentrations.

Under optimal experimental conditions, the calibration plot for detection by the proposed immunosensor with different concentrations of CD105 is illustrated in Figure 4. The cyclic voltammgram current responses of the resulting immunosensor after the antigen-antibody reaction increased with the increase of CD105 concentration in purified serum. The calibration curve for the membrane immunosensor under optimal experimental conditions showed a linear response to CD105. The concentration range was from 1.3 to 200.0 ng/mL with a detection limit of 0.9 ng/mL. A linear regression equation *i* = 0.4255 *C* + 74.845 and correlation coefficient of 0.9952 were obtained (Figure 4). A performance compared between the studied immunosensor and other conventional methods and some other similar immunosensors is shown in Table 1.

### 3.5. Specificity, Regeneration and Stability of the Sensor

The potential for selectivity is one of the main advantages of using biological molecules as recognition elements in biosensors. The effect of possible interference from other molecules on the response of the developed immunosensor was studied. The interference substances included carcinoembryonic antigen (CEA), human IgG, hepatitis B surface antigen, hepatitis B core antigen, hepatitis B surface antigen and α-fetoprotein. The degree of interference of the substance can be examined by detecting the amperometric responses. The imunosensors were separately exposed to 40 ng/mL CD105 solutions in purified serum with or without interferent. The responses of cyclic voltammgrams in the two solutions showed no remarkable differences, indicating that the proposed immunosensor based on the highly specific antigen-antibody immunoreaction had a good selectivity to CD105.

The stability of the successive assays was studied over 100 cycles of CV measurements in the working buffer after being incubated with 40 ng/mL of CD105. A 3.5% decrease of the initial response was observed. The long-term stability of the immunosensor was also investigated over a 60 days period. The immunosensor was stored at 4 °C and measured every 5 days. It was found that there were no apparent changes of the current response and 6.7% RSD was obtained

## 4. Conclusions

A novel immunosensor for the determination of CD105 with good sensitivity and high stability based on the PtNP-THI-Ab bioconjugates was described. The proposed immunosensor had several attractive advantages, such as high stability, easily adsorptive immobilization of antibody on AuNP monolayer, and the use of PtNP-THI-Ab bioconjugates to increase the sensitivity and selectivity of the immunosensor. Although the strategy has only been applied to anti-CD105 and CD105 as a model system, it could be readily extended toward the determination of other clinically or environmentally interested biospecies.

## Figures and Tables

**Table 1.** Performance compared with other conventional methods and immunosensor.

**Target Protein**	**Detection Methods**	**Linear Ranges**	**Detection Limits**	**Reference**
CD105	Cyclic voltammgram	1.3–200 ng/mL	0.9 ng/mL	Present work
CD105	ELISA	0.012–30 ng/mL	<0.01 ng/mL	[21]
CD105	RIA	15–225 ng/mL	--	[22]
Neomycin	Chronoamperometry	10–250 ng/mL	6.76 ng/mL	[23]
CEA	Cyclic voltammgram	0.5–10–60 ng/mL	0.4 ng/mL	[18]
